# Physical and Chemical Characteristics of Droppings as Sensitive Markers of Chicken Health Status

**DOI:** 10.3390/ani14091389

**Published:** 2024-05-06

**Authors:** Erika Mozuriene, Ernestas Mockus, Dovile Klupsaite, Vytaute Starkute, Ernesta Tolpeznikaite, Valentas Gruzauskas, Romas Gruzauskas, Agne Paulauskaite-Taraseviciene, Vidas Raudonis, Elena Bartkiene

**Affiliations:** 1 Institute of Animal Rearing Technologies, Faculty of Animal Sciences, Lithuanian University of Health Sciences, Mickeviciaus Str. 9, LT-44307 Kaunas, Lithuania; erika.mozuriene@lsmuni.lt (E.M.); ernestas.mockus@lsmuni.lt (E.M.); dovile.klupsaite@lsmuni.lt (D.K.); vytaute.starkute@lsmuni.lt (V.S.); ernesta.tolpeznikaite@lsmuni.lt (E.T.); 2 Department of Food Safety and Quality, Faculty of Veterinary Medicine, Lithuanian University of Health Sciences, Mickeviciaus Str. 9, LT-44307 Kaunas, Lithuania; 3 Artificial Intelligence Centre, Kaunas University of Technology, K. Barsausko 59, LT-51423 Kaunas, Lithuania; valentas.gruzauskas@ktu.lt (V.G.); romas.gruzauskas@ktu.lt (R.G.); agne.paulauskaite-taraseviciene@ktu.lt (A.P.-T.); vidas.raudonis@ktu.lt (V.R.)

**Keywords:** broilers, droppings, color parameters, short-chain fatty acids, volatile compounds

## Abstract

**Simple Summary:**

The characteristics of poultry droppings can reflect the health status of chickens. However, the existing literature lacks information on the physical–chemical properties of droppings, which could be useful for the development of practical and reliable diagnostic tools to monitor chickens’ welfare status. In order to expand the database in this field, this study examines the physical–chemical properties (e.g., texture, color, acidity, (short-chain) fatty acids, volatile compounds, etc.) of chicken droppings, which were collected during different chicken age periods (0–5, 6–10, 11–20, 21–30, and 31–40 days) and classified by visual inspection into normal and abnormal. The findings of this study show that normal droppings have a harder texture, less redness and yellowness, higher dry matter content, higher level of linoleic fatty acid, and lower level of α-linolenic fatty acid than abnormal ones in each age period. The age period of the chicken had a significant influence on most of the tested properties of the droppings. While some properties show that normal and abnormal droppings differ from one another, a presumably wider variety of droppings is needed to show more precise trends regarding the distribution of characteristics across normal and abnormal droppings of chickens at different ages.

**Abstract:**

The aim of this study was to analyze the physical and chemical characteristics of chicken droppings (n = 73), which were collected during different age periods and classified by visual inspection into normal (N) and abnormal (A). Significant differences were found in the texture, pH, dry matter (DM), fatty acids (FAs), short-chain fatty acids (SCFAs), and volatile compounds (VCs) between the tested dropping groups (*p* ≤ 0.05). The age period of the chicken had a significant influence on the color coordinates, texture, pH, DM, and SCFA contents in N and A as well as on all FAs content in N (*p* ≤ 0.05). Droppings from the N group had a harder texture, lower values of a* and b* color coordinates, higher DM content, higher level of linoleic FA, and lower level of α-linolenic FA than the droppings from the A group in each age period (*p* ≤ 0.05). The predominant SCFA was acetic acid, the content of which was significantly lower in the N group compared to that of the A group. The alcohol and organic acid contents were the highest in most of the A group at different age periods, while ketones dominated in the N and A groups. In conclusion, the majority of the tested dropping characteristics were influenced by the age period. While certain characteristics demonstrate differences between N and A, a likely broader range of droppings is required to provide more distinct trends regarding the distribution of characteristics across different droppings.

## 1. Introduction

The production of broilers is a significant aspect of the poultry business and supplies a substantial amount of the meat products sold worldwide [1]. Disease control becomes more difficult when broiler production intensifies in tandem with the rise in demand for animal products [2]. Given that a sizable amount of the world’s protein needs is supplied by the poultry sector, the worldwide rise in chicken illness has caused great alarm on a global scale [3]. A rapid and precise evaluation of the health of chickens can help producers make better decisions, lessen the spread of disease, enhance animal welfare, and maintain financial resources.

The intestinal barrier of the chicken contributes to its protection against pathogens, the colonization of commensal microorganisms, as well as the digestion and the absorption of nutrients [3,4]. This barrier consists of microbial, chemical, physical, and immunological factors distributed throughout different layers [5]. Gastrointestinal bacteria in poultry intestines ferment non-digestible carbohydrates to produce short-chain fatty acids (SCFAs) as energy, and it has been reported that acetate, propionate, and butyrate are the main metabolic products of the microbiome in poultry [6]. An important category of poultry diseases that has a detrimental impact on the economy and results in a substantial increase in morbidity and mortality are gastrointestinal issues [7]. Disturbances of the intestinal microbiota in poultry may affect the more vulnerable to infections, harming both their health and even the safety of the production [8]. Maintaining good health and growth for chickens in farming systems requires giving them appropriate nourishment. Nutrition and gut health are related, and the field of gut health is broad and encompasses immunology, microbiology, and physiology [9].

Chicken performance and welfare are greatly influenced by the daily management procedures used on farms too [10]. Typical techniques for evaluating the health of chickens involve collecting samples on the spot and then diagnosing the illness in a laboratory [11]. This process can take several days and calls for knowledgeable veterinarians and laboratory technicians. Additionally, on-site diagnosis of poultry diseases can be performed with fast detection kits, which may have a low detection rate for specific infections [12]. 

The health of chickens can be directly indicated by their droppings, which also serve as an important indicator of illness and digestive health. Potential intestine health issues brought on by bacterial, viral, or parasite diseases as well as nutritional inadequacies can be identified early on by examining the features of chicken droppings [13,14]. Currently, manual observation by veterinarians is used to examine unusual chicken droppings. Although this method is time-consuming and labor-intensive, the effective development and integration of other technologies, such as automated sensors or vision technologies, into the poultry production chain is still pending [14,15]. Although some farm management practices, such as visual assessment of dropping consistency for diarrhea severity in pig farms, offer a non-invasive method, these subjective scoring systems lack objectivity and consistency [16]. Therefore, research on specific biomarkers that can help identify the health condition of chickens is still needed. For example, bacteria can produce volatile organic compounds that are distinctive to them and may be employed in diagnostic procedures because these compounds can act as biological indicators of their presence [17]. On the contrary, the presence of SCFAs may indicate healthy gut microbiota [18]. Until now, the existing literature has lacked information on the physical and chemical parameters of normal and abnormal chicken droppings, which could be useful in biomarkers research and for the development of practical and reliable diagnostic tools for monitoring chicken welfare status.

The aim of this study was to analyze the physical and chemical characteristics of chicken droppings, which were collected during different age periods and classified by visual inspection into different categories (normal and abnormal). This was performed in order to better understand the age period influence on dropping characteristics and whether each category is reflected by differences in the physical and chemical characteristics of the droppings.

## 2. Materials and Methods

### 2.1. Samples Collection

Cecal dropping samples (n = 73) were collected from chickens aged 3–37 days. The keeping conditions and nutritional recommendation of the broiler chicken Ross 308 meet the requirements of Council Directive 2007/43/EC of 28 June 2007 [19,20,21,22]. All broiler chickens in the experiment had the same diet and housing conditions.

**Figure 1:**
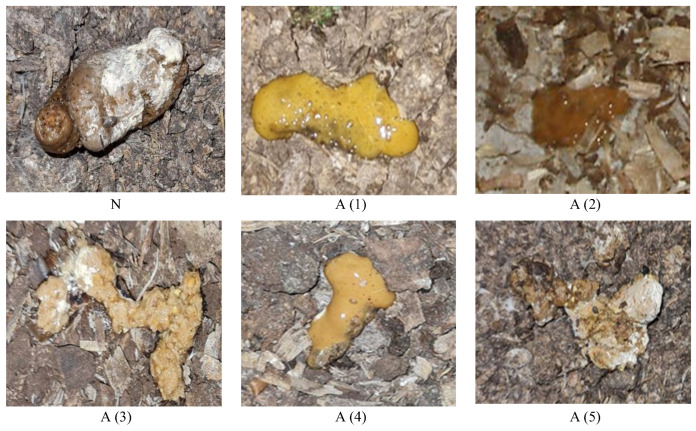
Images of droppings of broiler chickens. N—Normal; A—Abnormal; (1)—foamy; (2)—liquid, foamy; (3)—with feed residues; (4)—liquid; (5)—pathology.

Dropping samples were taken from deep litter in the different poultry houses. The collection of samples was performed in age periods of 0–5 days (I group), 6–10 days (II group), 11–20 days (III group), 21–30 days (IV group), and 31–40 days (V group) (Table 1). The visual appearance of droppings was evaluated by a poultry veterinarian, who used the Biomin-Feces catalog for the division of droppings into normal and abnormal groups. Images of chicken droppings are given in Figure 1. Samples were stored at −20 °C within labelled plastic tubes for further chemical analysis.

### 2.2. Evaluation of pH, Dry Matter, Texture, and Color Coordinates of Droppings

Using a pH meter (Inolab 3, Hanna Instruments, Villafranca Padovana PD, Italy), the samples’ pH was measured. After drying the droppings at 103 ± 2 °C to a consistent weight, the samples’ dry matter (DM) was calculated. The color coordinates were fixed at three different points of the sample surface using the CIE L*a*b* system (CromaMeter CR-400, Conica Minolta, Tokyo, Japan). Texture hardness was measured as the energy required for sample deformation (CT3 Texture Analyzer, Brookfield, Middleboro, MA, USA).

### 2.3. The Evaluation of Short-Chain Fatty Acids in Chicken Droppings

Short-chain fatty acids (SCFAs) (acetic acid, propanoic acid, isobutyric acid, butyric acid, isovaleric acid, valeric acid, and caproic acid) were determined as described by Zhao et al. [16] with some modifications, which are given in Supplementary File S1. Gas chromatography–mass spectrometry was used to analyze SCFAs.

### 2.4. Analysis of Volatile Compounds by Gas Chromatography–Mass Spectrometry

Samples for gas chromatography analysis were prepared by using solid phase microextraction (SPME). The detailed description of analysis is given in Supplementary File S1.

### 2.5. Fatty Acid Profile Analysis

The fatty acid profile of the dropping samples was determined using gas chromatography–flame ionization detection. The detailed description of the analysis is given in Supplementary File S1.

### 2.6. Statistical Analysis

All analytical analyses of the droppings were performed in triplicate. Shapiro–Wilk test was applied to test the normality of data. A two-way ANOVA, followed by the Tukey HSD post hoc test, was conducted to examine the effect of age period (I, II, III, IV, and V) and type of droppings (normal and abnormal) on physical and chemical characteristics of chicken droppings. The significance level was considered as *p* < 0.05. Data analysis was performed by the IBM SPSS Statistics 23.0 (Version 23.0, SPSS, Chicago, IL, USA).

## 3. Results and Discussion

### 3.1. pH, Dry Matter, Texture, and Color Coordinates of Chicken Droppings

The texture, pH, and dry matter (DM) of chicken droppings in tested sample groups are given in Table 2. It should be mentioned that normal droppings were detected in the I, II, III and IV age periods, while abnormal droppings were detected in the in II, III and IV age periods, and abnormal droppings with possible pathology were detected in the IV and V age periods. Significant differences were found in the analyzed parameters between all samples (*p* ≤ 0.05).

The results of the texture analysis show that the hardest sample was II GR N7, while the samples of I GR N4, II GR A7, III GR A13, III GR A14, and IV GR A27 had the softest texture. The texture of normal droppings significantly differed between all age periods (*p* ≤ 0.05). The texture of normal droppings was significantly harder than all abnormal droppings in age periods II, III, and IV (*p* ≤ 0.05). The texture of abnormal droppings was similar only between age periods II and III (*p* ≥ 0.05).

The highest pH was found in several abnormal droppings of III GR A13, III GR A16, and IV GR A21. The pH of normal droppings was similar between age periods I and IV; II and III; II and IV (*p* ≥ 0.05). The pH of abnormal droppings was similar between age periods III and IV (*p* ≥ 0.05). The same tendency was also found between age periods IV and V in abnormal droppings with possible pathology (*p* ≥ 0.05).

I GR N4, II GR N7, and III GR A13 had the highest DM content, while the lowest content was found in II GR A7, IV GR A27, and IV GR A27_1. The content of DM in normal droppings was significantly higher than that of abnormal ones in each age period (*p* ≤ 0.05). The DM of normal droppings significantly differed between age periods IV and II as well as between II and III (*p* ≤ 0.05). The DM of abnormal droppings was similar between age periods II and IV (*p* ≥ 0.05), while the III age period differed from the latter significantly (*p* ≤ 0.05). The DM of abnormal droppings with possible pathology significantly differed between age periods IV and V (*p* ≤ 0.05). The analysis of two-way ANOVA indicated that age period, type of droppings, and interaction between these effects were significant regarding the texture and dry matter content of the droppings (*p* < 0.001). Moreover, it was found that age period was a statistically significant factor for the pH of the droppings (*p* = 0.28).

Diets play a major role in the microbial variety of droppings, which results in modifications in the cecum [23]. Our results are similar to those found by Martínez et al. [24], who reported a pH of 6.54 for the droppings. Moreover, Jaramillo et al. [25] suggest that the pH variations in the droppings can be related to the type of volatile fatty acids of the diet. It is well known that organic acids stimulate pancreatic output. They can also acidify the digestive tract, lowering the pH of the gastrointestinal tract to 5.15. The increase in crude protein digestibility may be related to the proventriculus, the stomach of chickens, having an acidic composition [26].

The color coordinates of the chicken’ droppings are given in Table 3. It was observed that IV GR A21 had the highest values of lightness (L*), while the lowest L* was found in abnormal droppings of III GR A14 and III GR A16. The L* values of the normal droppings from the III age period significantly differed from those of the I, II, and IV age periods (*p* ≤ 0.05). These values were similar only between II and III age periods in abnormal droppings.

The highest redness (a*) was found for V GR A37 droppings, while the lowest in I GR N4 and II GR N7. In all cases, redness values were lower for normal droppings compared to abnormal ones in each age period. The a* values of the normal and abnormal (with possible pathology) droppings significantly differed between age periods (*p* ≤ 0.05). In abnormal droppings, the a* values of the IV age period significantly differed from those of the II and III age periods (*p* ≤ 0.05).

The yellowness (b*) of V GR A37 and V GR A36 was the highest, while the lowest b* values were found for I GR N4. In all cases, the b* values were lower for normal droppings compared to abnormal ones in each age period. The b* values of the normal droppings were only similar between the II and IV age periods. In abnormal droppings, the b* values from the III age period significantly differed from those of the II and IV age periods (*p* ≤ 0.05). The b* values of the abnormal (with possible pathology) droppings significantly differed between age periods (*p* ≤ 0.05). Analysis of two-way ANOVA indicated that age period was a statistically significant factor on the color coordinates L* and b* of the droppings (*p* = 0.06 and *p* < 0.001, respectively), while the color coordinate a* of the droppings was significantly influenced by age period and type of droppings (*p* < 0.001).

Season, chicken breed, and health condition affect the color, texture, and shape of droppings. The most effective method for identifying any abnormalities in the digestive tract is the droppings examination [27]. According to the observations of Li Guoming et al. [15] and Machuve et al. [28], a healthy bird excretes solid droppings with little liquid and are usually in shades of brown with a kind of small white covering on top [27]. A bird infected with Coccidiosis has fresh yellow watery or predominantly dark brown droppings with a flat shape [15,27], but some foods, like corn, strawberries, tomatoes, and forsythia flowers, can also cause it [27]. The droppings of a bird infected with Newcastle disease were fluid and mixed in color, appearing light yellow and green. Although the white hue may be misunderstood, the texture differs between healthy and salmonella-infected birds, appearing slimy and solid, respectively [15,28]. Greenish droppings can be caused by a diet high in vegetables, herbs, grass, weeds, and all types of plants, as well as intestinal worms, Marek’s illness, or bird flu. Black color droppings may occur from internal bleeding or from consuming charcoal, dark berries, or wood ash. Lastly, lead poisoning, coccidiosis, and intestinal wall edema or inflammation can all be the cause of orange or red droppings [27]. According to the results of this study, normal droppings possessed a harder texture, higher content of DM, and lower values of a* and b* color coordinates than abnormal droppings in each age period. As differences of texture, DM content, and values of a* and b* color coordinates were significant between normal and abnormal droppings in each age period, these characteristics could be used as sensitive markers for chicken health status identification.

### 3.2. Short-Chain Fatty Acid Profile of Chicken Droppings

The profile of short-chain fatty acids (SCFAs) in chicken droppings is given in Table 4. The percentage of SCFAs was significantly different between samples (*p* ≤ 0.05). The predominant SCFA in all samples was acetic acid with the highest content found in II GR A7 and V GR A37. The lowest content of acetic acid was found in normal droppings of III GR N13 and IV GR N22 as well as in abnormal droppings of III GR A13, III GR A16, and IV GR A21. Acetic acid content was significantly lower in normal droppings compared to abnormal droppings from the II and IV age periods (*p* ≤ 0.05). The acetic acid content of normal droppings significantly differed between all age periods (*p* ≤ 0.05), but it was similar only between the III and IV age periods in abnormal droppings.

The contents of acetic, butyric, and propanoic acid in abnormal droppings (with possible pathology) were similar between different age periods. Butyric acid was found in one normal and six abnormal dropping sample groups with the highest values in II GR A7 and III GR A14, and the lowest values in V GR A36. The butyric acid content in normal droppings of the I age period significantly differed from the rest (*p* ≤ 0.05). In abnormal droppings, the butyric acid content of the II age period significantly differed only from that of the IV age period (*p* ≤ 0.05).

Propanoic and isovaleric acids were found only in four sample groups of fourteen, while only two sample groups contained valeric acid. The content of these SCFAs was the highest in II GR A7. Moreover, in abnormal droppings, these SCFA values were similar only between the III and IV age periods. Low concentrations of isobutyric and caproic acid were only observed in II GR A7 and I GR N4, respectively. Analysis of two-way ANOVA indicated that age period, type of droppings, and interaction between these effects were statistically significant for the acetic, propanoic, isobutyric, butyric, isovaleric, and valeric acid contents in the droppings (*p* < 0.001).

The profile of SCFAs changes under the influence of numerous factors such as diet, external environment, health status, and the intestinal microbiota of the animal. The fermentation of dietary carbohydrates can yield SCFAs, such as acetic acid, propionic acid, and butyric acid. Additionally, some dietary protein and amino acids can ferment into SCFAs and lactic acid, and the levels of these acids can indicate the health of the gut microbiota and microbial activity [29]. Throughout a variety of commercial applications, the SCFAs—which are the most significant intermediate products of anaerobic digestion and include acetic acid, propionic acid, butyric acid, isobutyric acid, valeric acid, isovaleric acid, and caproic acid—have garnered growing attention [30]. Our results agree with those found by Mahato et al., 2022 [31], which found that, among the carboxylic acids, the acetic acid fraction was higher than those of the propionic and butyric acids in all tested chicken dropping samples [31,32,33]. These three substances make up 95% of all SCFAs and are primarily found in a 60/20/20 ratio, with acetic acid being the most prevalent [34]. The primary source of odorants is the anaerobic bacterial fermentation of volatile FAs, particularly acetic and propionic acids, which are created when proteins and amino acids degrade [12,35,36]. One of these odorants that is most prevalent in farm animal feces or manure is tryptophan metabolites produced by bacteria [37], and indole and skatole are regarded as the main contributors to the malodorous smell of animal excreta [38]. The SCFAs such as acetate, propionate, and butyrate are among the major metabolites from fermentation by gut commensals on undigested carbohydrates [39], while branched-chain FAs are found in undigested protein in the lower tract [40]. The SCFAs were studied as indicators of digestion and carbohydrate fermentation in avian caeca. Generally, the SCFA concentrations were higher in older birds and in bird groups fed with enzyme-supplemented diets [41]. Our results are in agreement with those found by Palander et al. 2020 [42], which found that, for all diets and ages, the concentration of acetic acid was the highest among the SCFAs, the proportion reaching from 58 to 76% of the total SCFAs.

### 3.3. Fatty Acid Profile of the Broiler Chicken Droppings

The FA profile of the broiler chicken droppings is given in Table 5. Analysis of two-way ANOVA indicated that age period and type of droppings were statistically significant for the oleic (C18:1), linoleic (C18:2 cis), and alfa linolenic acid (C18:3 α) contents in the droppings (*p* < 0.05). A significant effect of age period was also found for palmitic (C16:0), stearic (C18:0), and eicosenoic (C20:1) acid contents in the droppings (*p* = 0.03; *p* = 0.003; *p* < 0.001, respectively). There was a statistically significant interaction between the effects of age period and type of droppings on C16:0 content in the droppings (*p* = 0.04).

The predominant FAs were C18:1, C18:2 cis, and C18:3 α. The highest content of C18:1 was found in III GR A16, IV GR N22, and IV GR A27. The C18:1 content in normal droppings was similar only between the II and III age periods. However, the content of this FA was similar in all age periods in abnormal droppings (*p* ≥ 0.05), but significantly differed between age periods in abnormal droppings with possible pathology (*p* ≤ 0.05).

II GR N7, III GR N13, and IV GR A27_1 had the highest contents of C18:2 cis. The lowest content of this FA was found in II GR A7 and III GR A16. Normal droppings contained higher level of this FA than abnormal droppings in each age period. C18:3α was found at the highest level in II GR A7 and V GR A37, while IV GR N22 contained the lowest level of this FA. Normal droppings contained a lower level of C18:3α than abnormal droppings in each age period. In normal droppings, the contents of C18:2cis and C18:3α were similar only in the II and III age periods (*p* ≥ 0.05). The contents of C18:2cis and C18:3α in the abnormal droppings (and with possible pathology) were similar between different age periods.

Stearic (C18:0) and palmitic (C16:0) FAs were determined in lower levels than previously mentioned FAs, and all samples contained these FAs. IV GR A23 and V GR A36 had the highest level of C18:0 and V GR A36 also had the greatest level of C16:0. In normal droppings, the contents of C16:0 were similar only between the I and II as well as between the II and III age periods (*p* ≥ 0.05). The same tendency was observed for C18:0. In abnormal droppings, the C16:0 content of the III age period significantly differed from that of the IV age period (*p* ≤ 0.05). In the same droppings, the C18:0 content significantly differed between all age periods (*p* ≤ 0.05). The contents of both FAs were similar between different age periods in abnormal droppings with possible pathology.

Erucic (C22:1) and eicosadienoic (C20:2) FAs were only present in the normal droppings of I GR N4. γ—Gama linolenic (C18:3) acid was only found in the normal droppings of III GR N13, while arachidic acid (C20:0) was present only in the abnormal droppings of IV GR A21. Eicosenoic acid (C20:1) was found in one sample group of normal droppings (I GR N4) and two sample groups of abnormal droppings (II GR A7 and IV GR A21).

There is a lack of data concerning changes in the FA profile of chicken droppings at different age periods. A study on the droppings of Sprague Dawley rats showed that, although the FA compositions in different groups were different, the C16:0 and C18:0 were the dominant FAs in the droppings, followed by C18:1 and C18:2, while C18:3 was found in minor contents in all groups [43]. Another study on swine manure reported that the free FAs occurring in greatest abundance in fresh manure were palmitic, oleic, and stearic acids [44]. According to the results of this study, normal droppings contained a higher level of linoleic FA and a lower level of α-linolenic FA than abnormal droppings in each age period. As differences in C18:2 cis and C18:3α were significant between normal and abnormal droppings in each age period, these FAs could be used as sensitive markers for chicken health status identification.

### 3.4. Volatile Compound Profile of the Broiler Chicken Droppings

**Figure 2:**
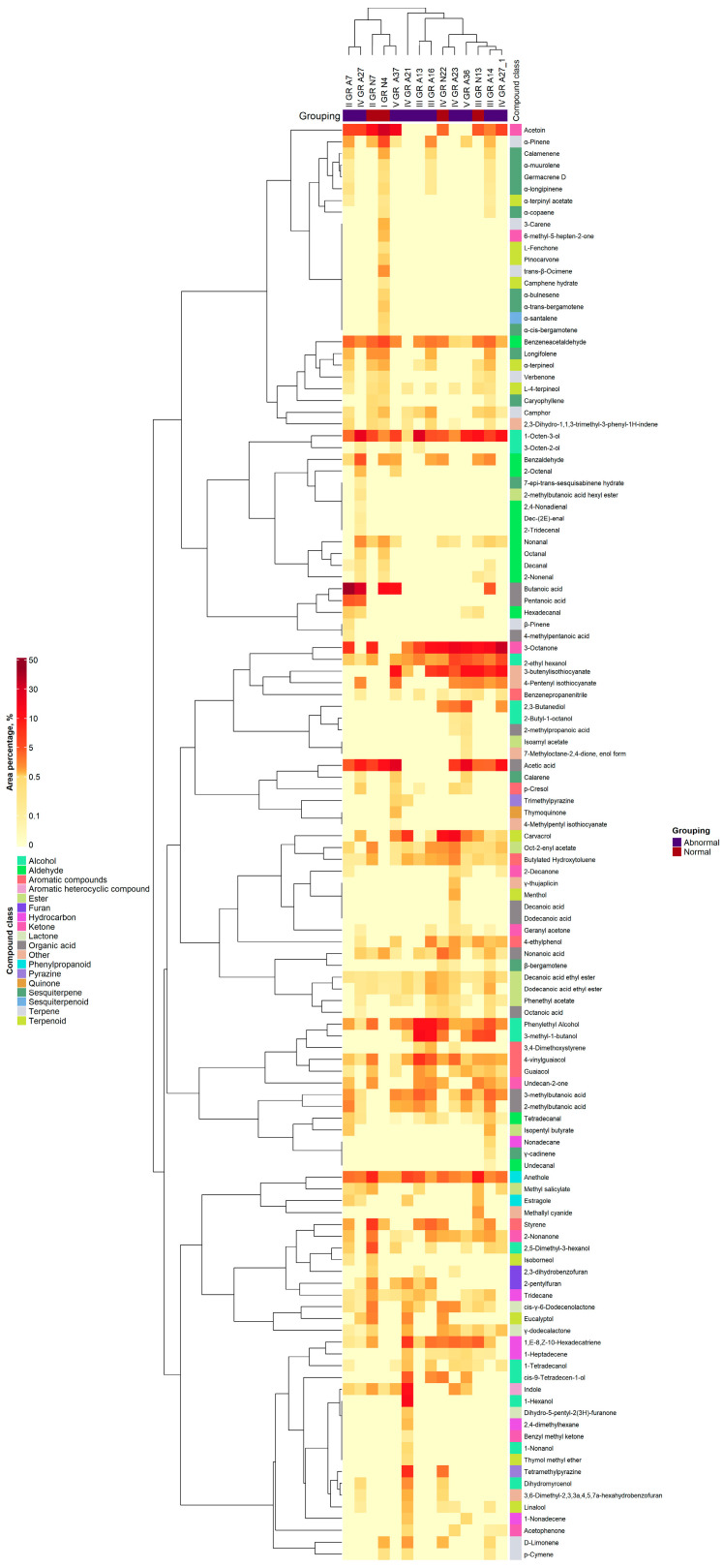
Volatile compound content (percentage from the total volatile compound content) in the droppings of broiler chickens.

The profile and concentration of volatile compounds (VCs) in chicken droppings by heatmap analysis are given in Figure 2. As shown in Figure 2, color coding was graded on the basis of the scale from light yellow to dark red with the relative intensity increasing from low (light yellow) to high (dark red).

The following groups of VCs were found in the tested droppings: thirteen alcohols and aldehydes, twelve sesquiterpenes and terpenoids, eleven organic acids, ten furans and terpenes, eight ketones and aromatic compounds, six hydrocarbons, three lactones, two phenylpropanoids and pyrazines as well as one aromatic heterocyclic compound, quinone and sesquiterpenoid. The VCs with the highest concentrations were alcohols, ketones, and organic acids, followed by aldehydes, aromatic compounds, phenylpropanoids, and terpenoids. The highest content (56.2%) of organic acids was found in the II GR A7 group. Moreover, these VCs were predominant in IV GR A27 and IV GR A37 (45.1 and 40.6%, respectively). Alcohols dominated in several abnormal dropping groups such as III GR A13 (60.3%), III GR A16 (34.2%), IV GR A21 (29.8%), and V GR A36 (24.0%). The highest content (41.7%) of ketones was found in IV GR A27_1 and these VCs dominated in all tested normal droppings of different age periods as well as in several other groups of abnormal droppings (III GR A14 and IV GR A23).

Concentrations of acetic acid, acetoin, 3-methyl-1-butanol, butanoic acid, 1-octen-3-ol, 3-butenyl isothiocyanate, 3-octanone, phenylethyl alcohol, indole, carvacrol, and 1-hexanol were the highest among other VCs (up to 42%). Their presence in different concentrations was observed in most of the samples, except 1-hexanol. However, clear tendencies regarding their distribution among the tested sample groups cannot be drawn. The acetic acid content was the highest in the V age period. Its content in normal droppings significantly differed between all age periods (*p* ≤ 0.05). The same tendency was observed for acetoin, except between the III and IV age periods. Its content was the highest in I GR N4, II GR N7, and V GR A37. The contents of 3-methyl-1-butanol and phenylethyl alcohol were the highest in the abnormal droppings of III GR A13 and III GR A16, while for butanoic acid they were the highest in II GR A7 and IV GR A27. The butanoic acid content in normal droppings of the I age period was significantly different from the rest (*p* ≤ 0.05). III GR A13 and IV GR A27 droppings contained a high level of 1-octen-3-ol, while IV GR N22 and IV GR A23 had the highest concentration of carvacrol. Analysis of two-way ANOVA indicated that age period, type of droppings, and interaction between these effects were statistically significant for the butanoic acid and carvacrol contents in the droppings (*p* < 0.001). Acetoin, 3-butenyl isothiocyanate, and 1-hexanol contents were significantly influenced by age period and type of droppings (except 1-hexanol) (*p* < 0.001). Age period and interaction between two factors were statistically significant for acetic acid, 3-methyl-1-butanol, and phenylethyl alcohol contents in the droppings (*p* < 0.001).

Odorous compounds, such as indole and SCFAs, are mostly produced in the droppings of broilers [29]. The presence of such common metabolic by-products as acetoin, acetic acid, butanoic acid and 1-octen-3-ol, 3-butenyl isothiocyanate, and indole in droppings is related to the decomposition of feed organic materials and fermentation processes by microorganisms that take place in the chickens’ digestive system [29]. Yasuhara [45] and Bicudo et al. [46] also observed similar compounds to ours in poultry and swine manure. It was reported that acetoin, which is an enzymatic decarboxylation product of pyruvate, can be produced by most *Bacillus bacteria*, which have been identified in duck droppings [47]. Butanoic acid has been linked to the maintenance of a healthy intestinal environment in chickens and, together with 3-butenyl isothiocyanate and indole, adds to the distinctive smell of chicken droppings [48]. 1-Octen-3-ol has been detected in the urine of elderly cattle and other terrestrial mammal emissions [49]. Fresh droppings may not contain 1-octen-3-ol, but it can become more noticeable as the droppings age and undergo microbial degradation because this compound is produced during the biodegradation of lipids by bacteria or fungi [50]. The presence of terpenoids in chicken droppings can be explained by the applied diet and (or) other forms of contamination such as during sample collection or from the farm environment [51].

3-Methyl-1-butanol, carvacrol, 1-hexanol, phenylethyl alcohol, and 3-octanone are not common constituents of chicken droppings, and some of them likely appear due to undigested feed components. An indicator of bacterial decline in chicken was found to be 3-methyl-1-butanol, which is generated by bacteria through the metabolism of valine and leucine [52]. Thus, some studies regarded 3-methyl-1-butanol as an indicator of bacterial deterioration in chickens [53]. Moreover, 3-methyl-1-butanol is a microbial VC frequently found in humid environments in agriculture and composting areas [50]. Carvacrol is mostly found in oregano oil [54]. 1-Hexanol, phenylethyl alcohol, and 3-octanone are naturally found in various plants, flowers, fruits, and vegetable essential oils, as well as beverages [55,56].

Methallyl cyanide, 6-methyl-5-hepten-2-one, 3-carene, trans-β-ocimene, L-fenchone, pinocarvone, α-bulnesene, α-bergamotene, and α-santalene were found in small concentrations (<2%) and only in normal droppings of I GR N4 and III GR N13 groups. Each compound was present only in one group of samples. Isopentyl butyrate was found in abnormal droppings of II GR A7 III and GR A14 III, while the presence of α-muurolene was observed in the mentioned samples also, as well as in III GR A16.

1-Hexanol, isoamyl acetate, 4-methylpentyl isothiocyanate, 1-nonanol, menthol, 7-methyloctane-2,4-dione, 2,4-nonadienal, hexyl 2-methylbutanoate, thymol methyl ether, thymoquinone, dec-(2E)-enal, thujaplicin, and dihydro-5-pentyl-2(3H)-furanone were present only in abnormal samples of the IV and V groups in low levels (<1.2%, except 1-hexanol). However, each compound was present only in one group of samples.

## 4. Conclusions

This study’s findings complement the lacking database about normal and abnormal chicken droppings’ physical and chemical properties, which may benefit studies of chicken health issues related to microbiota and could be further used by technology developers to create diagnostic tools for monitoring chickens’ welfare status. Age period, type of droppings, and interaction between these effects were significant for the texture, dry matter, and most of the SCFA contents of the droppings. Most fatty acid contents were significantly influenced by the age period and type of droppings, while age period significantly affected the acidity, lightness, and yellowness of the droppings. In every age period, normal droppings outperformed abnormal ones in terms of texture hardness, redness, yellowness, dry matter content, linoleic acid level, and α-linolenic acid level (*p* < 0.05). Although the volatile compound profile was broad and certain compounds were present in a small number of samples, clear tendencies regarding their distribution among dropping groups cannot be drawn. In general, age period influenced most of the characteristics of the droppings, and although certain characteristics indicate differences among normal and abnormal droppings, a probably broader spectrum of droppings is needed to provide clearer tendencies about the characteristic distribution between various droppings.

## Figures and Tables

**Table 1:** Description of sample groups.

Periods of Sample Collection	Group Name	Visual Droppings Evaluation	Explanation	Average Chicken Age
I GR (0–5 days)	I GR N4	Normal	Brown or greyish-brown, solid, with a tiny bit of covered white on top	4 days
II GR (6–10 days)	II GR N7	Normal	Brown or greyish-brown, solid, with a tiny bit of covered white on top	7 days
	II GR A7	Abnormal	Liquid	7 days
III GR (11–20 days)	III GR N13	Normal	Brown or greyish-brown, solid, with a tiny bit of covered white on top	13 days
	III GR A13	Abnormal	Mixed with feed residues	13 days
	III GR A14	Abnormal	Liquid	14 days
	III GR A16	Abnormal	Liquid/foamy	16 days
IV GR (21–30 days)	IV GR N22	Normal	Brown or greyish-brown, solid, with a tiny bit of covered white on top	22 days
	IV GR A23	Abnormal	Feed residues, possible pathology, coccidiosis, *Eimeria averculina*	23 days
	IV GR A27	Abnormal	Possible pathology, coccidiosis, *Eimeria acervulina*, *Eimeria maxima*	27 days
	IV GR A27_1	Abnormal	Feed residues, possible pathology, coccidiosis, *Eimeria maxima*	27 days
	IV GR A21	Abnormal	Mixed with feed residues	21 days
V GR (31–40 days)	V GR A37	Abnormal	Possible pathology, mixed with intestinal mucosa	37 days
	V GR A36	Abnormal	Possible pathology, mixed with feed residues	36 days

**Table 2:** Texture hardness, pH, and dry matter (DM) characteristics in the droppings of broiler chickens.

Sample Group Name	Texture Hardness, mJ	pH	DM, %
I GR N4	0.20 ± 0.01 a	5.10 ± 0.21 ab	35.36 ± 1.38 jk
II GR N7	0.70 ± 0.03 e	5.83 ± 0.23 adef	37.30 ± 1.46 k
II GR A7	0.20 ± 0.01 a	5.25 ± 0.21 adg	21.03 ± 0.82 a
III GR N13	0.50 ± 0.02 d	5.96 ± 0.24 eh	33.38 ± 1.30 ghij
III GR A13	0.20 ± 0.01 a	6.63 ± 0.27 hi	38.68 k ± 1.51
III GR A14	0.20 ± 0.01 a	5.83 ± 0.23 cdef	30.84 ± 1.20 fg
III GR A16	0.30 ± 0.01 b	6.90 ± 0.28 i	31.34 ± 1.22 fh
IV GR N22	0.40 ± 0.02 c	5.27 ± 0.21 ae	31.89 ± 1.24 fi
IV GR A23	0.30 ± 0.01 b	5.42 ± 0.22 afgh	26.14 ± 1.02 bd
IV GR A27	0.20 ± 0.01 a	4.84 ± 0.19 a	19.80 ± 0.77 a
IV GR A27_1	0.30 ± 0.01 b	5.70 ± 0.23 bceg	22.93 ± 0.89 ab
IV GR A21	0.40 ± 0.02 c	6.99 ± 0.28 i	26.79 ± 1.05 de
V GR A37	0.40 ± 0.02 c	5.74 ± 0.23 bcdef	28.88 ± 1.13 cdf
V GR A36	0.50 ± 0.02 d	5.21 ± 0.21 acg	25.64 ± 1.00 bce

Data are represented as means (n = 3) ± SD. a–k Means with different letters in column are significantly different (*p* ≤ 0.05). DM—dry matter; I, II, III, IV, V—age periods; GR—group; N—normal droppings; A—abnormal droppings; 4, 7, 13, 14, 16, 21, 22, 23, 27, 36, 37—average age (days) of broiler chickens.

**Table 3:** Color coordinates (L *, a *, b *) of chicken droppings.

Sample Group Name	Color Coordinates, NBS		
	L*	a*	b*
I GR N4	53.13 ± 2.26 ef	0.40 ± 0.02 a	5.75 ± 0.26 a
II GR N7	48.58 ± 2.06 cde	0.63 ± 0.02 ab	10.06 ± 0.45 cdef
II GR A7	41.89 ± 1.78 b	2.24 ± 0.09 d	9.00 ± 0.40 bc
III GR N13	42.46 ± 1.80 b	1.96 ± 0.08 d	11.92 ± 0.53 fg
III GR A13	41.10 ± 1.75 b	2.06 ± 0.08 d	11.61 ± 0.52 fg
III GR A14	33.77 ± 1.43 ab	3.18 ± 0.13 f	11.43 ± 0.51 fg
III GR A16	34.64 ± 1.47 ab	2.01 ± 0.08 d	9.40 ± 0.42 be
IV GR N22	52.65 ± 2.24 ef	0.75 ± 0.03 b	9.28 ± 0.41 bd
IV GR A23	39.66 ± 1.68 b	2.56 ± 0.10 e	11.90 ± 0.53 fg
IV GR A27	54.60 ± 2.32 f	1.46 ± 0.06 c	11.59 ± 0.52 fg
IV GR A27_1	45.46 ± 1.93 bd	3.14 ± 0.12 f	15.03 ± 0.67 h
IV GR A21	68.00 ± 2.89 g	1.20 ± 0.05 c	8.11 ± 0.36 b
V GR A37	43.07 ± 1.83 bc	4.68 ± 0.18 g	18.75 ± 0.84 i
V GR A36	49.56 ± 2.10 df	3.17 ± 0.13 f	20.36 ± 0.91 i

Data are represented as means (n = 3) ± SD. a–i Means with different letters in column are significantly different (*p* ≤ 0.05).; I, II, III, IV, V—age periods; GR—group; N—normal droppings; A—abnormal droppings; 4, 7, 13, 14, 16, 21, 22, 23, 27, 36, 37—average age (days) of broiler chickens; L*, lightness; a*, redness or −a*, greenness; b*, yellowness or −b*, blueness; NBS, National Bureau of Standards units.

**Table 4:** Short-chain fatty acids content (mmol/kg) in chicken droppings.

Sample Group Name	Acetic Acid	Propanoic Acid	Isobutyric Acid	Butyric Acid	Isovaleric Acid	Valeric Acid	Caproic Acid
I GR N4	26.07 ± 1.05 bc	nd	nd	1.71 ± 0.07 b	nd	nd	0.040 ± 0.002 a
II GR N7	22.73 ± 0.92 b	nd	nd	nd	nd	nd	nd
II GR A7	93.39 ± 3.78 h	5.41 ± 0.24 d	0.44 ± 0.02	16.50 ± 0.64 e	0.56 ± 0.02 d	0.75 ± 0.03 b	nd
III GR N13	10.11 ± 0.41 a	nd	nd	nd	nd	nd	nd
III GR A13	5.96 ± 0.24 a	nd	nd	nd	0.14 ± 0.01 b	nd	nd
III GR A14	62.70 ± 2.54 g	1.74 ± 0.08 b	nd	16.24 ± 0.63 e	nd	0.28 ± 0.01 a	nd
III GR A16	10.32 ± 0.42 a	0.83 ± 0.04 a	nd	2.09 ± 0.08 bc	0.28 ± 0.01 c	nd	nd
IV GR N22	5.99 ± 0.24 a	nd	nd	nd	nd	nd	nd
IV GR A23	37.64 ± 1.52 e	nd	nd	nd	nd	nd	nd
IV GR A27	45.10 ± 1.82 f	2.27 ± 0.10 c	nd	3.58 ± 0.14 d	nd	nd	nd
IV GR A27_1	32.65 ± 1.32 de	nd	nd	nd	nd	nd	nd
IV GR A21	5.46 ± 0.22 a	nd	nd	nd	0.066 ± 0.03 a	nd	nd
V GR A37	93.22 ± 3.77 h	nd	nd	2.55 ± 0.10 c	nd	nd	nd
V GR A36	28.45 ± 1.15 cd	nd	nd	0.59 ± 0.02 a	nd	nd	nd

Data are represented as means (n = 3) ± SD. a–h Means with different letters in column are significantly different (*p* ≤ 0.05).; I, II, III, IV, V—age periods; GR—group; N—normal droppings; A—abnormal droppings; 4, 7, 13, 14, 16, 21, 22, 23, 27, 36, 37—average age (days) of broiler chickens.

**Table 5:** Fatty acid content (percentage from the total fat content) in the droppings of broiler chickens.

Sample Group	C16:0	C18:0	C20:0	C18:1 *cis*	C20:1	C22:1	C18:2 cis	C20:2	C18:3 γ	C18:3 α
I GR N4	13.39 ± 0.53 g	5.03 ± 0.23 bd	nd	19.93 ± 0.86 ac	4.97 ± 0.23 b	1.10 ± 0.04	31.94 ± 1.11 c	0.69 ± 0.03	nd	22.96 ± 0.93 e
II GR N7	13.96 ± 0.56 gh	5.43 ± 0.25 cde	nd	17.59 ± 0.75 a	nd	nd	48.01 ± 1.67 g	nd	nd	15.00 ± 0.61 c
II GR A7	7.72 ± 0.31 ab	7.05 ± 0.32 hij	nd	24.35 ± 1.04 d	1.03 ± 0.05 a	nd	21.62 ± 0.75 a	nd	nd	38.23 ± 1.55 h
III GR N13	14.96 ± 0.60 hi	5.94 ± 0.27 ef	nd	17.25 ± 0.74 a	nd	nd	47.45 ± 1.65 g	nd	0.35 ± 0.01	14.05 ± 0.57 c
III GR A13	14.81 ± 0.59 gi	4.85 ± 0.22 bc	nd	23.84 ± 1.02 d	nd	nd	38.00 ± 1.32 def	nd	nd	18.50 ± 0.75 d
III GR A14	8.92 ± 0.36 bcd	4.64 ± 0.21 b	nd	17.14 ± 0.74 a	nd	nd	38.12 ± 1.32 def	nd	nd	31.18 ± 1.26 f
III GR A16	9.97 ± 0.40 de	6.07 ± 0.28 eg	nd	28.43 ± 1.22 e	nd	nd	20.66 ± 0.72 a	nd	nd	34.86 ± 1.41 g
IV GR N22	9.05 ± 0.36 bce	2.95 ± 0.14 a	nd	27.61 ± 1.18 e	nd	nd	55.60 ± 1.93 h	nd	nd	4.78 ± 0.19 a
IV GR A23	19.05 ± 0.76 j	7.15 ± 0.33 hik	nd	24.46 ± 1.05 d	nd	nd	27.76 ± 0.96 b	nd	nd	21.57 ± 0.87 e
IV GR A27	15.61 ± 0.62 i	6.66 ± 0.30 fgi	nd	28.41 ± 1.22 e	nd	nd	34.72 ± 1.20 cd	nd	nd	14.59 ± 0.59 c
IV GR A27_1	8.11 ± 0.32 ac	2.66 ± 0.12 a	nd	31.49 ± 1.35 f	nd	nd	49.57 ± 1.72 g	nd	nd	8.17 ± 0.33 b
IV GR A21	6.86 ± 0.27 a	2.27 ± 0.10 a	0.14 ± 0.01	19.67 ± 0.84 ab	5.43 ± 0.25 c	nd	35.04 ± 1.22 cf	nd	nd	30.59 ± 1.24 f
V GR A37	11.64 ± 0.47 f	6.63 ± 0.30 fgh	nd	20.58 ± 0.88 bc	nd	nd	25.80 ± 0.90 b	nd	nd	35.35 ± 1.43 gh
V GR A36	20.56 ± 0.82 j	7.51 ± 0.34 jk	nd	26.24 ± 1.13 bc	nd	nd	34.76 ± 1.21 ce	nd	nd	10.92 ± 0.44 b

Data are represented as means (n = 3) ± SD. a–k Means with different letters in column are significantly different (*p* ≤ 0.05).; I, II, III, IV, V—age periods; GR—group; N—normal droppings; A—abnormal droppings; 4, 7, 13, 14, 16, 21, 22, 23, 27, 36, 37—average age (days) of broiler chickens; C16:0—palmitic acid; C18:0—stearic acid; C18:1—oleic acid; C18:2 cis—linoleic acid; C18:3 α—alfa linolenic acid; C18:3 γ—gama linolenic acid; C20:1—eicosenoic acid; C20:2—eicosadienoic acid; C22:1—erucic acid; C20:0—arachidic acid.

## Data Availability

Dataset available upon request from the authors.

## Supplementary Materials

The following supporting information can be downloaded at: https://www.mdpi.com/article/10.3390/ani14091389/s1.

## References

[1] Maharjan P., Martinez D.A., Weil J., Suesuttajit N., Umberson C., Mullenix G., Hilton K.M., Beitia A., Coon C.N. (2021). Review: Physiological Growth Trend of Current Meat Broilers and Dietary Protein and Energy Management Approaches for Sustainable Broiler Production. Animal.

[2] Thi Huong-Anh N., Van Chinh D., Thi Tuyet-Hanh T. (2020). Antibiotic Residues in Chickens and Farmers’ Knowledge of Their Use in Tay Ninh Province, Vietnam, in 2017. Asia Pac. J. Public Health.

[3] Hossain M.S., Salsabil U.S., Syeed M.M., Rahman M.M., Fatema K., Uddin M.F. SmartPoultry: Early Detection of Poultry Disease from Smartphone Captured Fecal Image. Proceedings of the 2023 20th International Joint Conference on Computer Science and Software Engineering (JCSSE).

[4] Duangnumsawang Y., Zentek J., Goodarzi Boroojeni F. (2021). Development and Functional Properties of Intestinal Mucus Layer in Poultry. Front. Immunol..

[5] Wickramasuriya S.S., Park I., Lee K., Lee Y., Kim W.H., Nam H., Lillehoj H.S. (2022). Role of Physiology, Immunity, Microbiota, and Infectious Diseases in the Gut Health of Poultry. Vaccines.

[6] Waite D.W., Taylor M. (2015). Exploring the Avian Gut Microbiota: Current Trends and Future Directions. Front. Microbiol..

[7] Borgonovo F., Ferrante V., Grilli G., Guarino M. (2024). An Innovative Approach for Analysing and Evaluating Enteric Diseases in Poultry Farm. Acta IMEKO.

[8] Corrigan A., de Leeuw M., Penaud-Frézet S., Dimova D., Murphy R.A. (2015). Phylogenetic and Functional Alterations in Bacterial Community Compositions in Broiler Ceca as a Result of Mannan Oligosaccharide Supplementation. Appl. Environ. Microbiol..

[9] Jha R., Singh A.K., Yadav S., Berrocoso J.F.D., Mishra B. (2019). Early Nutrition Programming (in Ovo and Post-Hatch Feeding) as a Strategy to Modulate Gut Health of Poultry. Front. Vet. Sci..

[10] van Veen L.A., van den Oever A.C.M., Kemp B., van den Brand H. (2023). Perception of Laying Hen Farmers, Poultry Veterinarians, and Poultry Experts Regarding Sensor-Based Continuous Monitoring of Laying Hen Health and Welfare. Poult. Sci..

[11] Vidic J., Manzano M., Chang C.-M., Jaffrezic-Renault N. (2017). Advanced Biosensors for Detection of Pathogens Related to Livestock and Poultry. Vet. Res..

[12] Cho S., Hwang O., Park S. (2015). Effect of Dietary Protein Levels on Composition of Odorous Compounds and Bacterial Ecology in Pig Manure. Asian-Australas. J. Anim. Sci..

[13] He P., Wu R., Liu D., Dou J., Hayat K., Shang D., Pan J., Lin H. (2024). An Efficient Segmentation Model for Abnormal Chicken Droppings Recognition Based on Improved Deep Dual-Resolution Network. J. Anim. Sci..

[14] Nakrosis A., Paulauskaite-Taraseviciene A., Raudonis V., Narusis I., Gruzauskas V., Gruzauskas R., Lagzdinyte-Budnike I. (2023). Towards Early Poultry Health Prediction through Non-Invasive and Computer Vision-Based Dropping Classification. Animals.

[15] Li G., Gates R.S., Ramirez B.C. (2023). An On-Site Feces Image Classifier System for Chicken Health Assessment: A Proof of Concept. Appl. Eng. Agric..

[16] Pérez-Calvo E., Wicaksono A.N., Canet E., Daulton E., Ens W., Hoeller U., Verlhac V., Celi P., Covington J.A. (2019). The Measurement of Volatile Organic Compounds in Faeces of Piglets as a Tool to Assess Gastrointestinal Functionality. Biosyst. Eng..

[17] Bos L.D.J., Sterk P.J., Schultz M.J. (2013). Volatile Metabolites of Pathogens: A Systematic Review. PLoS Pathog..

[18] Fusco W., Lorenzo M.B., Cintoni M., Porcari S., Rinninella E., Kaitsas F., Lener E., Mele M.C., Gasbarrini A., Collado M.C. (2023). Short-Chain Fatty-Acid-Producing Bacteria: Key Components of the Human Gut Microbiota. Nutrients.

[19] EUR-Lex Official Journal of the European Union, L:2007:182:TOC—EN. https://eur-lex.europa.eu/legal-content/EN/TXT/?uri=OJ%3AL%3A2007%3A182%3ATOC.

[20] EUR-Lex Official Journal of the European Union Regulation—EU-2017/625-of the European Parliament and of the Council—EN. https://eur-lex.europa.eu/eli/reg/2017/625/oj.

[21] (2018). Managment Book Ross Broiler, Managment Handbook. https://aviagen.com/assets/Tech_Center/Ross_Broiler/Ross-BroilerHandbook2018-EN.pdf.

[22] Ross 300 PS, Managment Handbook. http://www.rosspoultrybreeders.co.za/downloads/breeder/2018RossPSHandbook.pdf.

[23] Oakley B.B., Lillehoj H.S., Kogut M.H., Kim W.K., Maurer J.J., Pedroso A., Lee M.D., Collett S.R., Johnson T.J., Cox N.A. (2014). The Chicken Gastrointestinal Microbiome. FEMS Microbiol. Lett..

[24] Martínez Y., Altamirano E., Ortega V., Paz P., Valdivié M. (2021). Effect of Age on the Immune and Visceral Organ Weights and Cecal Traits in Modern Broilers. Animals.

[25] Jaramillo Á. (2012). Evaluation of a Prebiotic and an Organic Acid-Supplemented Diets on the Performance and Allometric Pa-Rameters of Broiler Chickens with Controlled Feeding. Rev. Colomb. Cienc. Pecu..

[26] Ndelekwute E.K., Unah U.L., Udoh U.H. (2019). Effect of Dietary Organic Acids on Nutrient Digestibility, Faecal Moisture, Digesta pH and Viscosity of Broiler Chickens. MOJ Anat. Physiol..

[27] Damerow G. (2016). The Chicken Health Handbook: A Complete Guide to Maximizing Flock Health and Dealing with Disease.

[28] Machuve D., Nwankwo E., Mduma N., Mbelwa J. (2022). Poultry Diseases Diagnostics Models Using Deep Learning. Front. Artif. Intell..

[29] Zhu X., Tao L., Liu H., Yang G. (2023). Effects of Fermented Feed on Growth Performance, Immune Organ Indices, Serum Biochemical Parameters, Cecal Odorous Compound Production, and the Microbiota Community in Broilers. Poult. Sci..

[30] Wainaina S., Lukitawesa, Kumar Awasthi M., Taherzadeh M.J. (2019). Bioengineering of Anaerobic Digestion for Volatile Fatty Acids, Hydrogen or Methane Production: A Critical Review. Bioengineered.

[31] Mahato P., Rajagopal R., Goyette B., Adhikary S. (2022). Low-Temperature Anaerobic Digestion of Chicken Manure at High Organic and Nitrogen Loads—Strategies for Controlling Short Chain Fatty Acids. Bioresour. Technol..

[32] Díaz-Corona L.R., Parra-Saavedra K.J., Mora-Alonzo R.S., Macías-Rodríguez M.E., Martínez-Preciado A.H., Guevara-Martínez S.J., Zamudio-Ojeda A., Macias-Lamas A.M. (2023). HPLC-DAD Development and Validation Method for Short-Chain Fatty Acids Quantification from Chicken Feces by Solid-Phase Extraction. Separations.

[33] Ali Q., Ma S., La S., Guo Z., Liu B., Gao Z., Farooq U., Wang Z., Zhu X., Cui Y. (2022). Microbial Short-Chain Fatty Acids: A Bridge between Dietary Fibers and Poultry Gut Health—A Review. Anim. Biosci..

[34] den Besten G., van Eunen K., Groen A.K., Venema K., Reijngoud D.-J., Bakker B.M. (2013). The Role of Short-Chain Fatty Acids in the Interplay between Diet, Gut Microbiota, and Host Energy Metabolism. J. Lipid Res..

[35] Tampio E.A., Blasco L., Vainio M.M., Kahala M.M., Rasi S.E. (2019). Volatile Fatty Acids (VFAs) and Methane from Food Waste and Cow Slurry: Comparison of Biogas and VFA Fermentation Processes. GCB Bioenergy.

[36] Yin J., Yu X., Wang K., Shen D. (2016). Acidogenic Fermentation of the Main Substrates of Food Waste to Produce Volatile Fatty Acids. Int. J. Hydrogen Energy.

[37] Liu H.Y., Li X., Zhu X., Dong W.G., Yang G.Q. (2021). Soybean Oligosaccharides Attenuate Odour Compounds in Excreta by Modulating the Caecal Microbiota in Broilers. Animal.

[38] Zhu X., Zhang Y., Liu H., Yang G., Li L. (2023). Microbiome-Metabolomics Analysis Reveals Abatement Effects of Itaconic Acid on Odorous Compound Production in Arbor Acre Broilers. BMC Microbiol..

[39] Peng Q., Zeng X.F., Zhu J.L., Wang S., Liu X.T., Hou C.L., Thacker P.A., Qiao S.Y. (2016). Effects of Dietary Lactobacillus Plantarum B1 on Growth Performance, Intestinal Microbiota, and Short Chain Fatty Acid Profiles in Broiler Chickens. Poult. Sci..

[40] Qaisrani S.N., Moquet P.C.A., van Krimpen M.M., Kwakkel R.P., Verstegen M.W.A., Hendriks W.H. (2014). Protein Source and Dietary Structure Influence Growth Performance, Gut Morphology, and Hindgut Fermentation Characteristics in Broilers. Poult. Sci..

[41] Palander S. Volatile Fatty Acid Profile in Caecal Digesta of Growing Turkey Poults.

[42] Palander S., Näsi M., Järvinen S. (2005). Effect of Age of Growing Turkeys on Digesta Viscosity and Nutrient Digestibility of Maize, Wheat, Barley and Oats Fed as Such or with Enzyme Supplementation. Arch. Anim. Nutr..

[43] Ye Z., Xu Y.-J., Liu Y. (2021). Influence of Different Dietary Oil Consumption on Nutrient Malabsorption: An Animal Trial Using Sprague Dawley Rats. J. Food Biochem..

[44] Loughrin J.H., Szogi A.A. (2006). Free Fatty Acids and Sterols in Swine Manure. J. Environ. Sci. Health B.

[45] Yasuhara A. (1987). Identification of Volatile Compounds in Poultry Manure by Gas Chromatography—Mass Spectrometry. J. Chromatogr. A.

[46] Bicudo J.R., Schmidt D.R., Powers W., Zahn J.A., Tengman C.L., Clanton C.J., Jacobson L.D. (2002). Odor and VOC Emissions from Swine Manure Storages. Proceedings of the Odors and Air Pollutants Conference 2002.

[47] Kimball B.A., Yamazaki K., Kohler D., Bowen R.A., Muth J.P., Opiekun M., Beauchamp G.K. (2013). Avian Influenza Infection Alters Fecal Odor in Mallards. PLoS ONE.

[48] Melaku M., Zhong R., Han H., Wan F., Yi B., Zhang H. (2021). Butyric and Citric Acids and Their Salts in Poultry Nutrition: Effects on Gut Health and Intestinal Microbiota. Int. J. Mol. Sci..

[49] Tangtrakulwanich K., Albuquerque T.A., Brewer G.J., Baxendale F.P., Zurek L., Miller D.N., Taylor D.B., Friesen K.A., Zhu J.J. (2015). Behavioural Responses of Stable Flies to Cattle Manure Slurry Associated Odourants. Med. Vet. Entomol..

[50] Ernstgård L., Norbäck D., Nordquist T., Wieslander G., Wålinder R., Johanson G. (2013). Acute Effects of Exposure to Vapors of 3-Methyl-1-Butanol in Humans. Indoor Air.

[51] Joguet N., Jing L., Jamois F., Dumargue P. (2023). Characterization of Volatile Organic Compounds (VOCs) from Farms Effluents: Interest of HS-SPME-GC-MS Technique for Laboratory and Field Test. Atmosphere.

[52] Al-Dalali S., Li C., Xu B. (2021). Effect of Frozen Storage on the Lipid Oxidation, Protein Oxidation, and Flavor Profile of Marinated Raw Beef Meat. Food Chem..

[53] Klein D., Maurer S., Herbert U., Kreyenschmidt J., Kaul P. (2018). Detection of Volatile Organic Compounds Arising from Chicken Breast Filets Under Modified Atmosphere Packaging Using TD-GC/MS. Food Anal. Methods.

[54] Alagawany M. (2015). Biological Effects and Modes of Action of Carvacrol in Animal and Poultry Production and Health—A Review. Adv. Anim. Vet. Sci..

[55] Sirilun S., Chaiyasut C., Sivamaruthi B.S., Peerajan S., Kumar N., Kesika P. (2017). Phenethyl Alcohol Is an Effective Non-Traditional Preservative Agent for Cosmetic Preparations. Asian J. Pharm. Clin. Res..

[56] Kyoui D., Saito Y., Takahashi A., Tanaka G., Yoshida R., Maegaki Y., Kawarai T., Ogihara H., Suzuki C. (2023). Antibacterial Activity of Hexanol Vapor In Vitro and on the Surface of Vegetables. Foods.

