# Supplementary material for: Physical and Chemical Characteristics of Droppings as Sensitive Markers of Chicken Health Status
Source: Animals (Basel). 2024 May 6;14(9):1389. doi: 10.3390/ani14091389 (PMC11083088; doi:10.3390/ani14091389)
Supplement: Supplementary file 1 [file animals-14-01389-s001.zip › animals-2973658-supplementary.pdf]

1. The Evaluation of Short-chain Fatty Acids in chicken droppings

Short-chain fatty acids (SCFAs) (acetic acid, propanoic acid, isobutyric acid, butyric acid, isovaleric acid, valeric acid, and caproic acid) were determined as described by Zhao et al. [16] with some modifications, which are given in Supplementary File S1. Sample (1 g) and 10 mL of water were homogenized for about 3 min. The pH of the obtained solution was adjusted to 2–3 with 5 M HCl and then kept at room temperature for 10 min during occasional shaking. Then the solution was centrifuged for 20 min at 5000 rpm, giving a clear supernatant. 1μl of the supernatant was injected in the Gas Chromatograph GC-2010 Plus (Shimadzu corp., Kyoto, Japan) with a Mass Spectrometer GCMS-QP2010 (Shimadzu corp., Kyoto, Japan) for analysis.

2. Analysis of Volatile Compounds by Gas Chromatography–Mass Spectrometry

Samples for gas chromatography (GC) analysis were prepared by using solid phase microextraction (SPME). A solid phase microextraction device with Stableflex (TM) fibre, coated with a 50-μm DVB-PDMS-Carboxen™ layer (Supelco, Bellefonte, PA, USA), was used for sample preparation. For headspace extraction, 1 g of sample in the 20-mL extraction vial, sealed with a polytetrafluoroethylene septum, was thermostated at 60 °C for 15 min, thereby exposing the fibre in the headspace. The fibre was then exposed to the headspace of the vial for a further 10 min. The desorption time was 2 min. For gas chromatography–mass spectrometry (GC–MS), a GCMS-QP2010 (Shimadzu, Japan) was used. The gas chromatograph was equipped with an AOC-5000 Plus Shimadzu autosampler, upgraded with an SPME analysis kit. The following analysis parameters were used: for the ionisation of analytes at 70 eV; for separation of VCs, a low-polarity Rxi®-5MS column (Restek, Bellefonte, PA, USA); injector temperature 250 °C, ion source, and interface temperatures 220 °C and 280 °C, respectively; the temperature gradient: from 35 °C (5 min hold) to 200 °C (10 °C/min) and then up to 280 °C (25 °C/min) (5 min hold); carrier gas was helium (99.999% detector purity, Linde, Vilnius, Lithuania), pressure of 6.61 psi (45.6 kPa); column flow 0.97 mL/min; the VC were identified according to the mass spectra libraries (NIST11, NIST11S, FFNSC2).

3. Fatty Acid Profile Analysis

The fatty acid (FA) profile composition of the dropping samples was determined using gas chromatography–flame ionisation detection (GC–FID; Agilent 6890N Gas Chromatograph, Agilent Technologies, Santa Clara, CA, USA). First, FA were dissolved in cyclohexane (100 mg in 4 mL), then methyl esters of FA were prepared by transmethylation using 8 mL of 1.5% sulphuric acid in methanol, and kept at 60 °C for 12 h. The samples were cooled, shaken for 30 s, and then centrifuged for 10 min at 3000× *g* at 17 °C. They were then injected with 100 μL of the upper portion of the supernatant (previously diluted with cyclohexane in the ratio of 1:9) into a capillary BPX90 column (60 m × 0.32 mm ID × 0.25-μm film thickness; SGE, Santa Clara, CA, USA). The following parameters were used: flame ionisation detector: 280 °C; H_2 flow: 40 mL/min; air flow: 450 mL/min; helium (carrier gas) flow: 1 mL/min; injector: 250 °C (split 1:10); oven temperature: 50 °C for 2 min, then 4 °C/min to 245 °C, and then 245 °C for 15 min. Each FA was identified according to its retention time and is expressed as a percentage of the total peak area of all FA in the sample.
